# Wild and Domestic Animal Exposure among Deceased Persons Referred for Organ Procurement, United States

**DOI:** 10.3201/eid3112.251486

**Published:** 2025-12

**Authors:** David W. McCormick, Raymond Lynch, Brianna Doby, Sarah Laskey, Pallavi Annambhotla, Ryan M. Wallace, Sarah C. Bonaparte, Lauri A. Hicks, Sridhar V. Basavaraju, Ian Kracalik

**Affiliations:** Centers for Disease Control and Prevention, Atlanta, Georgia, USA (D.W. McCormick, P. Annambhotla, R.M. Wallace, S.C. Bonaparte, L.A. Hicks, S.V. Basavaraju, I. Kracalik); Health Resources and Services Administration, Rockville, Maryland, USA (R. Lynch, B. Doby, S. Laskey)

**Keywords:** rabies, viruses, organ donation, transplantation, zoonoses, animal bites, animal scratches, United States

## Abstract

Rabies is transmissible through transplantation. Wild mammal bites or scratches carry a high risk for rabies, but their frequency among organ donors is unknown. During 2024, an estimated 12 (95% CI 7–20; 0.07%) of 16,989 deceased US donors had high-risk exposures. Identifying such exposures can mitigate rabies transmission to transplant recipients.

Rabies is a rare and nearly universally fatal infectious disease, primarily transmitted through the saliva of an infected mammal. Human-to-human transmission of rabies is rare and usually is associated with organ or tissue transplantation (*1*–*3*). In January 2025, the 7th US death from transplant-transmitted rabies since 1978 was reported in a kidney transplant recipient (*4*). The organ donor had a documented skunk exposure, prompting a national review of transplant-associated rabies risk. Additional screening of potential donors with animal bites or scratches could help mitigate the risk for rabies disease transmitted by organ transplantation. Wild animal exposures confer higher risk for rabies than domestic animal exposure (*5*). We estimated the annual number of US organ donors who had mammal bites, scratches, or both.

Next of kin provide information on prospective donors during a required deceased donor risk assessment interview (DRAI). Seven organ procurement organizations (OPOs) serving rural and urban settings in 7 of the 10 US Department of Health and Human Services regions (regions 2, 3, 4, 6, 8, 9, and 10) provided data on animal exposures collected using the DRAI for 4,555 persons referred for organ procurement during 2024 (Table). We defined an animal exposure as receipt of a bite or scratch within 12 months before referral for organ procurement. We determined the need for postexposure prophylaxis (PEP) among transplant recipients by using an online risk stratification tool (https://kellycharniga.shinyapps.io/RabiesRiskTool) to assess whether rabies PEP was indicated for recipients of organs from donors with wild mammal exposures (*5*).

**Table:** Exposures to wild and domestic mammals (bites or scratches) among persons referred for organ procurement from seven organ procurement organizations, United States, 2024*

Exposure type	No. (%)	
	Persons ruled in for organ procurement, n = 2,872	Persons ruled out for organ procurement, n = 1,683
Mammal†	151 (5.3)	90 (5.3)
Domestic‡	149 (5.2)	88 (5.2)
Cat	71 (2.5)	46 (2.7)
Dog	53 (1.8)	48 (2.9)
Livestock	1 (0.03)	2 (0.1)
Ferret	0	1 (0.06)
Rabbit	2 (0.07)	0
Sugar glider§	1 (0.03)	0
Pet or NOS	1 (0.03)	1 (0.06)
Wild‡	2 (0.07)	2 (0.1)
Rat	2 (0.07)	0
Porcupine	0	1 (0.06)
Raccoon	0	1 (0.06)
Bat	0	0

*NOS, not otherwise specified. †Respondents could report >1 animal exposure for each person referred for organ donation. ‡The Centers for Disease Control and Prevention–maintained National Respiratory and Enteric Virus Surveillance System collected 2024 animal rabies testing data from state and territorial public health departments and US Department of Agriculture Wildlife Services. Data from this system showed that <1.5% of domestic animals submitted for testing have rabies. §This animal (*Petaurus breviceps*) is a small, nocturnal, arboreal gliding possum that is kept as an exotic pet.

We categorized persons referred for organ procurement who meet suitability criteria as ruled-in persons. Not all persons referred for organ procurement meet suitability criteria for donation, and persons who do not meet those criteria do not become organ donors. We defined persons referred for organ procurement but who did not meet other suitability criteria as ruled-out persons. The overall national number of persons ruled in for organ procurement from whom organs are not procured is unknown.

Among 4,555 referred persons, 244 (5.4%) had a documented animal exposure, of which 4 (0.09%) were wild mammals (2 rats, 1 raccoon, and 1 porcupine). Of the 4,555 referred persons, 2,872 (63.1%) were ruled in for organ procurement by the OPOs after medical review, of whom 154 (5.4%) had a documented animal exposure, which is comparable to the proportion of animal exposures among persons ruled out for donation (90/1,683 [5.3%]) (Table). Because rabies only affects mammals, we excluded 3 nonmammalian animal exposures (parakeet, spider, and wasp).

Overall, among the 2,872 persons ruled in for organ donation, 151 (5.3%) had an animal exposure, of which 149 (98.7%) were domestic mammals and 2 (1.3%) were wild mammals (both rats). PEP was indicated for the porcupine and raccoon exposures in persons ruled out. The circumstances in which rabies PEP would be indicated for a bite or scratch from a rat would depend on whether the rat was provoked or unprovoked and if it was apparently healthy, ill, or acting strangely (*5*).

Extrapolating the findings from the 2,872 ruled-in persons to 16,989 deceased donors in 2024 (*6*), we estimated that 881 (5.2% [95% CI 825–938]) deceased donors would have a domestic mammal exposure and an additional 12 (0.07% [95% CI 7–20]) deceased donors would have a wild mammal exposure annually. On the basis of those estimates, domestic mammal exposures were common among deceased donors in the United States, whereas wild mammal exposures were rare. In our analysis, we estimated 12 deceased donors have wild mammal exposures annually, although those exposures might not be recognized at the time of procurement or be identified in the DRAI because next of kin might have poor recall. The frequency of unrecognized animal exposure, including exposure to bats (*7*), cannot be estimated by our analysis. Feral cats are the most frequently recognized source of rabies among domestic animals (*8**,**9*). Although no reports of exposure to feral cats among referred donors were in the provided data, exposure to feral cats should prompt further rabies assessment among persons referred for organ procurement.

Animal exposure is not a contraindication to organ procurement; however, prior transmission events demonstrate that donor exposures to wild mammals carry a risk for rabies to recipients and warrant further preventive interventions (*1*–*3*). Prompt administration of PEP can prevent rabies in transplant recipients (*1*–*3*). When high-risk animal exposure is identified in an organ donor, OPOs should consider contacting infectious disease specialists and public health officials to assess rabies risk and determine whether PEP or other mitigation strategies are indicated among recipients. Such measures are critical to mitigating potential transplant-associated rabies transmission.
